# Visual Impairment does not Limit Training Effects in Development of Aerobic and Anaerobic Capacity in Tandem Cyclists

**DOI:** 10.1515/hukin-2015-0095

**Published:** 2015-01-12

**Authors:** Kamelska Anna Malwina, Mazurek Krzysztof, Zmijewski Piotr

**Affiliations:** 1 Jozef Rusiecki Institute of Higher Education, Olsztyn Poland; 2 Department of Sports Medicine, Jozef Pilsudski University of Physical Education in Warsaw, Poland; 3 Department of Physiology, Institute of Sport, Warsaw Poland

**Keywords:** adults, athletes, cycling, exercise, fitness test, physiological profile

## Abstract

The study aimed to investigate the differences in the effects of 7-month training on aerobic and anaerobic capacity in tandem cycling athletes with and without visual impairment. In this study, Polish elite (n=13) and sub-elite (n=13) visually impaired (VI) (n=13; 40.8 ±12.8 years) and properly sighted (PS) (n=13; 36.7 ±12.2 years) tandem-cycling athletes participated voluntarily in 7-month routine training. The following pre-/post-training measurements were conducted on separate days: maximal oxygen uptake (VO_2max_) was estimated with age correction using the Physical Working Capacity test on a bicycle ergometer according to the Astrand-Ryhming method. Maximal power output (P_max_) was evaluated using the Quebec test on a bicycle ergometer. At baseline, VO_2max_ (47.8 ±14.1 vs 42.0 ±8.3 ml/kg/min, respectively) and P_max_ (11.5 ±1.5 vs 11.5 ±1.0 W/kg) did not differ significantly between PS and VI cyclists. However, differences in aerobic capacity were considered as clinically significant. Two-way ANOVA revealed that after 7 month training, there were statistically significant increases in VO_2max_ (p=0.003) and P_max_ (p=0.009) among VI (VO_2max_, +9.1%; P_max_, +6.3%) and PS (VO_2max_, +9.1%; P_max_, +11.7%) cyclists, however, no time × visual impairment interaction effect was found (VO_2max_, p=0.467; P_max_, p=0.364). After training, VO_2max_ (p=0.03), but not P_max_ (p=0.13), was significantly greater in elite compared to sub-elite tandem cyclists. VI and PS tandem cyclists showed similar rates of improvement in VO_2max_ and P_max_ after 7-month training. VO_2max_ was a significant determinant of success in tandem cycling. This is one of the first studies providing reference values for aerobic and anaerobic capacity in visually impaired cyclists.

## Introduction

Regular physical activity (PA) is beneficial for physical and psychological health, as it reduces the risk of heart diseases, obesity and diabetes, selected cancers, as well as clinical depression (Akbarpour, 2013; Lee et al., 2012; Warburton et al., 2006). Physical inactivity and low physical fitness were also established as independent predictors for cardiovascular diseases (CVD) (Dogra and Stathokostas, 2012). Despite that, the level of physical activity in developing countries is low. Physical inactivity is now identified as the fourth leading risk factor for global mortality (WHO, 2010). Individuals with visual impairment have consistently exhibited lower physical fitness and physical activity levels than the properly sighted children, youth and adults (Aslan et al., 2012; Marmeleira et al., 2014).

Visual impairment is one of the most common disabilities. There are different levels of this dysfunction. Moderate visual impairment is grouped together with severe visual impairment under the term ‘low vision’. Low vision and blindness represent all visual impairment according to the International Classification of Diseases-10 (WHO, 2006). The number of people of all ages visually impaired worldwide in 2012 was estimated to be 285 million, of whom 39 million were blind (Pascolini and Mariotti, 2012). Other reports have shown that 32.4 million people around the world are blind and 191 million people have moderate or severe vision impairment (Stevens et al., 2013). It is believed that without intervention, the number of the blind could increase to 76 million by 2020, mainly due to the rapid aging of populations in most countries (US Bureau of the Census, 1998).

Visual impairment and sensory defects result in difficulty in physical movement, efficient movement, and safety concerns for physical exercises (Chen and Lin, 2011). As a consequence, the visually impaired demonstrate difficulties of dynamic and static balance, poor posture, deficient motor coordination, impaired mobility and gait, and inappropriate muscle tone (Castelli Correia De Campos et al., 2013). This could lead to lower ability to perform various physical exercises.

Many visually impaired individuals do not voluntarily participate in sport activities. Children and adults who are blind have low levels of physical activity and are considerably less active compared with the general population (Holbrook et al., 2009; Marmeleira et al., 2014). The level of physical fitness for the visually impaired is generally lower and frequency of obesity is higher compared with the general population (Greguol et al., 2014). It was also reported that participation in moderate-to-vigorous physical activity (MVPA) was significantly higher in non-visually impaired individuals than in visually impaired individuals (Houwen et al., 2009). Participation in sports is one solution to involve individuals in MVPA. Health promotion strategies including sport activities should be implemented to increase daily PA for people with visual impairment (Marmeleira et al., 2014).

For visually impaired individuals, sports participation is also an opportunity to increase social interactions, thus, inclusion in the social life. The visually impaired student-athletes differed significantly from their non-athlete counterparts in socialization. Indeed, the athletes scored significantly higher than non-athletes on the socialization test (Movahedi et al., 2011). Sport can be used as a method to reduce and overcome disadvantages related to disabilities. Previous reports suggest that visually impaired individuals who participate in regular sport activities improve their fitness and demonstrate a level of fitness comparable to properly sighted ones (Lieberman et al., 2012; Williams et al., 1996). Tandem cycling is one form of exercise that can be used as part of rehabilitation. Tandem cycling is a current summer Olympic event in which individuals with visual impairment can officially participate (Wilson and Clayton, 2010).

Traditional cycling and tandem cycling are quite similar. However, cycling on a tandem results in lower physiological stress than when cycling at the same velocity on a single bicycle. Cyclists are able to ride faster on a tandem than on a single bicycle at similar physiological stress. Here, stokers can add to power output on a tandem without adding significantly to wind resistance (Seifert et al., 2003). Despite the growing popularity of tandem cycling, little is known about the tandem cyclists, training achievements and physiological responses to cycling on a tandem compared to a single bicycle in adults with visual impairment. The coaches have a great responsibility and need to have knowledge of training models, quantities of effort, capabilities of disabled athletes and other elements influencing health and exercise performance. Therefore, the present study aimed to investigate the differences in the effects of 7-month training on aerobic and anaerobic capacity in tandem cycling athletes with and without visual impairment.

## Material and Methods

### Participants

Twenty-six Polish elite (n=13) and sub-elite (n=13) tandem-cycling athletes participated in this study. The study group involved 13 visually impaired (VI) (40.8 ±12.8 years) and 13 properly sighted (PS) (36.7 ±12.2 years) athletes, who volunteered to take part in the research and 7-month routine training.

The visually impaired group (VI) consisted of functionally sighted athletes of different tandem-cycling national teams from Poland and of the Polish Blind Society. Properly sighted participants were the partners in tandem with visually impaired athletes. Athletes were matched for age and training experience.

The inclusion criteria were as follows: visual impairment (visual acuity V=0.05-0.3) which precluded independent cycling; no medical contraindications to participate in the research; and male sex. Exclusion criteria were as follows: the coexistence of other disabilities and diseases, and taking medications that could affect the results of the analyses.

Based on peak power output, power to weight ratios, and an average number of training hours per week, the subjects were regarded as well-trained cyclists (Jeukendrup et al., 2000). All of them were professional cyclists and had trained for at least five years.

### Experimental procedure

All subjects gave written informed consent to participate in this study. The study protocol was approved by the Senate Research Ethical Committee (number SKE 001-27/2012). The procedures were explained to the subjects both verbally and in writing (including in Braille).

The study protocol conformed to the ethical guidelines of the 1975 Declaration of Helsinki as reflected in a priori approval by the institution’s human research committee and followed the APA Ethics Standard.

The measurements were performed before (in the middle of the transition period) and after 7-month routine cycling training (at the beginning of the competitive period). The following pre-/post-training measurements were conducted on separate days: maximal oxygen uptake (VO_2max_) was estimated with age correction using the Physical Working Capacity test on a bicycle ergometer (Monark 874E, Sweden) according to the Astrand-Ryhming method. Maximal power output (P_max_) was evaluated using the Quebec test on a Monark 828 E.

All experiments were conducted between 9 am and 3 pm to ensure that the subjects had their normal amount of sleep (ranging between 6 and 9 hours per night) and time to eat their normal race day diet. The tests were conducted in a temperature-controlled room with a fan and were supported by two paramedics in case of emergency. Athletes had a light meal at least two hours and a maximum of four hours before the exercise test. No alcohol was allowed during the preceding 24 hours and no tobacco during the preceding 12 hours. From two hours before the first test and throughout the test session, the only type of fluid intake allowed was water.

### Training

The observation started in the middle of the transition period, which lasted for 2–3 months (detraining recovery phase). Endurance training included a 1.5 h workout 3 times a week (cycling, running, swimming).

The preparatory period lasted 3–4 months. In this period, the athletes participated in endurance training and general conditioning training sessions. Endurance training consisted of field workouts, in the form of cycling on a road tandem, in natural conditions, 3–4 times a week for a 2 h workout. Athletes trained alone as well as with partners with the intensity in the range of 75–80% HR_max_ (140–165 beats/min). During this period, when bad weather conditions occurred, athletes performed endurance training, e.g. running, or used a cycling ergometer. Moreover, general conditioning training consisted of sessions based on team games, gymnastics and weightlifting and was conducted approximately twice a week.

The competitive period lasted about 3 months. The volume and intensity of training during this period increased to 5 endurance training sessions per week (2–2.5 h each). Participation in racing competitions (over a distance of 40–110 km) at least once a month was introduced.

The structure of tandem-cycling athletes’ training was set by club coaches and was not changed due to the research. Moreover, the cyclists were instructed to continue to train and not deviate from their planned schedules during the course of the investigation. However, they were instructed to rest or only train at low intensity during the 24 hours preceding the tests. None of the cyclists had a sudden increase or decrease in training volume or intensity (for the last month) or suffered from illness (for the last month) or injury (for the last 2 months) upon entering the study.

### Measurements

#### Anthropometrics

Before the physiological tests were performed, anthropometric measurements were obtained. Subjects were dressed in underwear without shoes. Body height (BH) and mass (BM) were recorded using a standard portable column scale and a balance weighing scale (Tanita BC 545 MA, Tokyo, Japan), respectively. The body mass index (BMI) was calculated using the standard formula: body mass (kg)/body height^2^ (m). Body composition was determined by bioelectrical impedance analysis using the Tanita BC-545. In accordance with the manufacturer’s instructions, the subjects stood on the metal footpads with bare feet, and fat mass (%FAT) was determined using the prediction equations supplied by the manufacturer. Fat mass (kg) and fat-free mass (%FFM) were subsequently calculated from body mass using the measured fat mass (%FAT).

#### Physical capacity

Aerobic capacity was evaluated by an indirect method, using the PWC test (Physical Working Capacity) (Council of Europe, 1993) on a bicycle ergometer (Ergomedic 874E, Monark, Sweden) according to the Astrand-Ryhming method (Astrand and Rodahl, 1986; Noonan and Dean, 2000). The PWC test was based on two 3 min standard efforts on a Monark cycloergometer 828 E (Monark Exercise AB, Sweden), with an individually assigned load. The test was preceded by a 5 min warm-up at an initial workload of 1 W/kg body mass. The heart rate was measured every 15 s using an impedance cardiography method (Sodolski and Kutarski, 2007). The PWC index was calculated from the mean HR values recorded at the end of each effort. The PWC index was used to calculate maximal oxygen uptake (VO_2max_) from the Karpman formula (Karpman, 1969) with age correction. Relative PWC 170 was calculated as PWC 170 divided by body mass.

On separate days from the aerobic capacity test, each subject underwent an anaerobic test (AnT) which was performed on a mechanically braked cycle ergometer (Ergomedic 824E, Monark, Sweden) according to the procedures of the Quebec test (McArdle et al., 2010). The testing session started with a standardized 5 min warm-up of cycling at a self-selected intensity not exceeding approximately 50% of VO_2max_, and after a 5 min rest the AnT began with a load of 7.5% body mass (BM). The participants were instructed to accelerate to their maximal pedaling rate and verbally encouraged to maintain this pedaling cadence as long as possible throughout the 10 s test. Computer software (MCE V 5.1., JBA Staniak, Poland) automatically calculated peak power (PP), defined as the highest mechanical power expressed in W/kg of body mass.

### Statistics

The main effects of time (within-subject), group (between-subject), and their interactions on dependent variables were assessed via 2-factor ANOVA with time as a repeated measure. Subjects’ metric age was noted and incorporated into the statistical analysis as a covariate. The main effect of time was considered to be the effect of training and the main effect of group as the effect of disability. The magnitude of change after training, or difference between groups (VI vs. PS), was computed as the difference between the means, M1–M2, divided by the standard deviation (Cohen’s d effect size, ES). Threshold values for Cohen ES statistics were 0.2 (small), 0.5 (moderate) and 0.8 (large) (Cohen, 1988). Cohen (1988) suggested that the effect size may be the most meaningful analysis for comparing data obtained from small, uneven groups. Post-training values were used for analysis of differences between sub-elite and elite athletes. These values were evaluated with the Student’s t test or the Mann-Whitney U test, as appropriate. Statistical significance was set at *p*<0.05. Data processing and statistical evaluations were completed using SPSS version 19.0 for Windows (SPSS Inc, Chicago, IL).

## Results

At baseline, no significant differences were found between the VI and PS groups for any of the somatic and physical capacity variables measured before intervention.

The results of two-factor ANOVA for somatic variables revealed a significant time (training) effect (p<0.001); lower body mass and fat mass as well as greater fat-free mass were noted following the training period. No statistically significant interaction effect (time × group) was found for any of the somatic variables (Table 1).

Two-factor ANOVA for absolute and relative P_max_ revealed a significant time (training) effect (p=0.015 and p=0.002, respectively); higher P_max_ [W] and P_max_ [W/kg] values were noted after the training period (Table 1). A time × group interaction effect was found as insignificant for anaerobic power indices.

The results of two-factor ANOVA for aerobic capacity revealed a significant time (training) effect for absolute and relative VO_2max_ values (p=0.003 and p=0.012, respectively); both indices were greater following the training period. No statistically significant interaction effect (time × group) was found for aerobic capacity (Table 1).

Post-training values were used for analysis of differences between sub-elite and elite athletes as determinants of success in tandem cycling. The analysis revealed that anaerobic capacity indices (absolute and relative P_max_) did not differ significantly between sub-elite and elite athletes. However, Cohen’s d effect size was 0.57 for P_max_ [W] and 0.56 for P_max_ [W/kg] (Table 2).

Absolute VO_2max_ [l/min] and relative VO_2max_ [ml/kg/min] were significantly higher (p=0.04 and p=0.04) in elite than sub-elite athletes, and Cohen’s d effect size was 0.76 and 0.93 respectively (Table 2).

## Discussion

This study provides the first evidence regarding the physiological effect of long-term training in tandem cyclists. The key contribution of this study is to provide reference values of aerobic and anaerobic capacity for Polish tandem cyclists and describe possible training-induced changes in physical capacity. The literature review found no cycling training response studies related to the exercise rehabilitation process in visually impaired individuals. This research begins to fill the gap in knowledge. One of the most important findings is that visual impairment does not limit training effects in development of aerobic and anaerobic capacity in tandem cyclists.

The athletes participating in this study were typical of tandem cyclists (personal communication with the National Team’s coach). They were older than regular competitive cyclists or triathletes and had a greater BMI and fat mass (Brunkhorst and Kielstein, 2013; Menaspà et al., 2012), however, the observed values of body composition are regarded as normal, are lower than the mean values in the general population, and could be considered as beneficial for health (Islami et al., 2011). In a Catalonian study, visually impaired athletes showed similar anthropometric data to other athletes, normal in regard to the BMI, but oriented towards a predominance of the mesomorph somatotype (Torralba et al., 2015). It was found that athletes who were blind (B1) had lower physical fitness than the visually impaired (B2), which is an important issue for planning training and goals (Lieberman and McHugh, 2010; Torralba et al., 2015).

The mean VO_2max_ of the study sample was 49.0 ml/kg/min, which is categorized as above average based on gender and age (Grigaliūnienė et al., 2013). Our athletes possessed an average VO_2max_ (in L/min) of 3.63, which is much lower than reported data concerning professional cyclists (Lucía et al., 1998; Menaspà et al., 2010; Mujika and Padilla, 2001; Sallet et al., 2006; Tanaka et al., 1993). We did not observe significant differences in aerobic and anaerobic capacity between visually impaired and properly sighted cyclists. Many researchers have reported that visually impaired people exhibited lower fitness levels than sighted peers (Houwen et al., 2009; Lieberman and McHugh, 2010). Some researchers found no significant differences in VO_2max_ between low vision and sighted girls (Williams et al., 1996). Previous studies aimed at evaluating physical fitness of the visually impaired are limited. Liberman and McHugh (2010) pointed out that in the previous studies many different methods had been used to assess cardiovascular endurance, muscular strength, endurance and body composition, and the degree of visual impairment of the participants varied widely among the studies, what makes comparisons of the results problematic. The results of other studies are not consistent. Previously, it was demonstrated that totally blind subjects were less fit than sighted peers at least partly owing to their lower level of habitual activity (Hopkins et al., 1987). In non-athletes, blind individuals were significantly less active than able-bodied individuals, and the difference between mean VO_2max_ for blinded and normal subjects became non-significant when their different activity levels were taken into account (Hopkins et al., 1987). Other authors found no significant differences in VO_2max_, forced vital capacity, leg strength and power between the blind and the control groups involved in regular recreational activities (Singh and Singh, 1993). The results showed that the visually impaired subjects who were active could have similar levels of physical fitness, lung function and explosive leg strength as those of their active sighted counterparts (Singh and Singh, 1993). This is in agreement with findings from another study indicating that low physical work capacity in visually handicapped boys and young male adults is due to the lack of physical activity, and that mild endurance training is effective in improving physical and psychological symptoms as well as cardiorespiratory fitness (Shindo et al., 1987). Furthermore, physical fitness of visually impaired goalball players was even higher than that of the more sedentary group (p<0.05), except shoulder-stretch test values (p>0.05). It was considered that directing visually impaired individuals to participation in sports or recreational activities was important in improving their physical fitness (Karakaya et al., 2009). This is consistent with data reported by other authors, who found that levels of physical fitness of students in schools dedicated for students with visual impairment are higher than those of students from mainstream schools (Short and Winnick, 1986). Our data are also ambiguous; at baseline, we did not find significant differences between VI and PS athletes, either in body mass and composition or in physical capacity. However, one should note the great variability of results of aerobic capacity measurement. The participants in the present study were all competitive cyclists, but may be characterized as a heterogeneous performance group. PS cyclists had greater mean VO_2max_ (47.8 ml/kg/min) than VI by 5.8 ml/kg/min. This difference is relevant and should influence performance (Currell and Jeukendrup, 2008; Storen et al., 2013). Cohen’s d effect size was 0.50 for the group comparison and indicated a moderate effect of the visual disability; this allows us to conclude that the difference in VO_2max_ was relevant for performance.

We found that within 7 months of structured training there was a significant decrease in athletes’ body mass and improved body composition. It could be assumed that athletes were involved in strenuous endurance training and this enhanced the potential for increasing fatty acid oxidation. This is generally in agreement with other studies (Romijn et al., 1993). We also observed a significant effect of training on aerobic and anaerobic capacity in both groups. Absolute and relative VO_2max_ increased by 8.3% and 7.7% in properly sighted and 9.2% and 9.0% in visually impaired subjects, and this reflects the effectiveness of training. Our results are similar to the results reported by other researchers. Sassi et al. (2008) found within-subject changes in professional cyclists, reporting an 8.4% and 10.5% increase in absolute and relative VO_2max_ after 6 months of training (from the resting period to the competitive period). In professional cyclists within-subject variability in VO_2max_ could be smaller during the annual training cycle. Czuba and Zajac (2012) reported a 3.7% increase of VO_2max_ after 3 months of training. Lucia et al. (2000) analyzed the longitudinal changes over the course of a whole season in professional cyclists. They reported significant changes in ventilatory and lactate thresholds following pre-season winter training, whereas during the season aerobic fitness indices remained stable (Lucía et al., 2000). The different rates of VO_2max_ changes can be explained by the training programs (form, intensity, frequency and duration), the initial level, as well as age and sex of the subjects. Recently, Stroen et al. (2012) reported that a preseason reduced total training volume but an increased amount of high aerobic intensity interval running training greatly improved VO_2max_ and time trial performance without any changes in metabolic cost of cycling.

We did not observe significant training × group effects on physical capacity. The training-induced improvements in absolute and relative VO_2max_ (ml/kg/min) as well as in absolute and relative P_max_ were similar in both groups. These findings emphasized that visual impairment did not limit training effects in development of aerobic and anaerobic capacity in VI individuals. This confirms that in conditions dedicated for the visually impaired, significant improvements in physical fitness can be expected. This finding supports the hypothesis that in appropriate conditions impaired athletes could maintain the same physical fitness level as properly sighted persons (Lieberman et al., 2012).

The secondary objective of this study was to identify physiological and anthropometric determinants that could be used as predictors of performance in tandem cyclists, similarly to able-bodied cyclists. None of the anthropometric variables significantly correlated with performance, which is in accordance with some previous reports (Mujika and Padilla, 2001; Storen et al., 2013). Some authors reported a small effect of anthropometric characteristics on the performance level in cyclists (Menaspà et al., 2012). We found significant differences in values of absolute and relative VO_2max_ between elite and sub-elite tandem cyclist groups. This finding is consistent with the literature: it was previously demonstrated that among highly-trained cyclists only the aerobic characteristics could discriminate the performance level, with the absolute values being useful to discern among high, medium and low levels of cycling performance (Menaspà et al., 2012). In our study, Cohen’s d effect size value for comparison of these groups indicates moderate and strong effects for absolute and relative values of VO_2max_, respectively. Surprisingly, relative VO_2max_ was able to better discriminate than absolute values among different performance levels.

We did not find significant differences in anaerobic indices between elite and sub-elite cyclists. In some previous studies it was found that the sensitivity of the anaerobic all-out test did not allow other differentiations between elite and professional cyclists (Sallet et al., 2006). However, other researchers found that the better cyclists were characterized by higher aerobic and anaerobic power output (Tanaka et al., 1993). Anaerobic power values are sometimes recognized as important components associated with cycle performance in competitive cyclists (Baron, 2001). Again, our results of measuring P_max_ ([W] and [W/kg]) were highly variable within analyzed groups. After analyzing the effect size with Cohen’s d values (which is dedicated for analysis of small samples), we found an effect size which could be considered at least as moderate. This means that anaerobic capacity should not be undervalued, as a relevant determinant of success in tandem cycling. Previously, Storen et al. (2013) presented an equation showing that both aerobic and anaerobic endurance capacity indices correlated strongly with time trial laboratory performance (r = 0.93, p < 0.01, SEE = 5.7%). However, none of the strength, power or anthropometric variables correlated significantly with time trial laboratory performance (Storen et al., 2013). They also reported that even with a large set of physiological and anthropometrical variables only VO_2max_ and metabolic cost of cycling and the combination of these 2 variables seemed to be able to explain substantially part of the inter-individual differences in time trial laboratory performance. Nevertheless, the importance of anaerobic endurance capacity could increase if the performance was shorter. In cyclists anaerobic capacity and power decline significantly across the age spectrum with no change in aerobic capacity (Gent and Norton, 2013).

## Limitations

The participants in this study were heterogeneous in terms of disability, however, there was only a small number of elite cyclists with physical disabilities. Although there were no statistically significant differences in this study between the VI and PS tandem cyclists for the various performance and anthropometric measures, it should not be assumed that this would be the case in a larger sample. The small sample size could have affected the analysis. Future studies could possibly include continuous gas exchange measurement during the discipline-specific test. Further research should be designed to explore other training modalities to determine the most effective training process in this specific group.

To our knowledge, this is the first study showing effects of seasonal training-induced changes in aerobic and anaerobic capacity of visually impaired and sighted tandem cyclists. We found that visual impairment did not limit training effects in development of aerobic and anaerobic capacity in tandem cyclists. Our results indicated that cycling training was effective in improving physical factors. The current findings are consistent with previous studies in able-bodied cyclists, but have now for the first time been illustrated in cyclists with physical disabilities. Secondly, we confirmed that VO_2max_ alone is a significant determinant of success in tandem cycling and tandem road cyclists are classifiable. This is important to properly interpret the results of laboratory assessments and set training loads. These results might be useful to develop specific training programs and enhance performance of tandem cyclists.

## Figures and Tables

**Table 1:** Effects of 7-month cycling training on somatics, anaerobic and aerobic capacity in properly sighted and visually impaired tandem cyclists

	Properly sighted (n=13)		Visually impaired (n=13)		Training effect	Training × group interaction effect
	Pre	Post	Pre	Post		
Body mass [kg]	77.3 ± 8.2	75.6 ± 7.6	74.3 ± 8.8	72.7 ± 8.0	p=0.001	p=0.847
BMI	24.0 ± 2.7	23.4 ± 2.4	23.7 ± 1.9	23.2 ± 1.8	p=0.001	p=0.827
FM [%]	13.7 ± 3.7	12.3 ± 2.9	13.3 ± 1.8	12.1 ± 2.5	p=0.001	p=0.742
FFM [%]	86.3 ± 3.7	88.0 ± 3.2	86.9 ± 2.1	88.2 ± 3.1	p=0.001	p=0.640
TBW [%]	63.2 ± 2.7	64.3 ± 2.1	63.6 ± 1.5	65.1 ± 2.5	p=0.005	p=0.610
P_max_ [W]	891 ± 134	967 ± 150	852 ± 97	882 ± 122	p=0.015	p=0.267
P_max_ [W/kg]	11.5 ± 1.5	12.9 ± 2.2	11.5 ± 1	12.2 ± 1.9	p=0.002	p=0.269
VO_2max_ [l/min]	3.62 ± 1.01	3.92 ± 0.71	3.10 ± 0.73	3.34 ± 0.66	p=0.003^	p=0.392^
VO_2max_ [ml/kg/min]	47.8 ± 14.1	52.2 ± 9.8	42.0 ± 8.3	45.8 ± 6.9	p=0.012^	p=0.467^

BMI – body mass index, FM – fat mass, FFM – fat-free mass, TBW – total body water, P_max_ – maximal power output, VO_2max_ – maximal oxygen uptake,

^ analyzed with age as a co-variant

**Table 2:** Anaerobic and aerobic capacity in properly sighted and visually impaired sub-elite and elite tandem cyclists

	Tandem cyclists groups		Level of advance effect
	Sub-elite (n=13)	Elite (n=13)	
P_max_ [W]	885 ± 149	963 ± 126	p=0.33
P_max_ [W/kg]	12.0 ± 2.3	13.1 ± 1.6	p=0.41
VO_2max_ [l/min]	3.36 ± 0.57	3.89 ± 0.81	p=0.04
VO_2max_ [ml/kg/min]	45.2 ± 6.5	52.8 ± 9.6	p=0.04

P_max_ – maximal power output, VO_2max_ – maximal oxygen uptake
